# The predictive effect of subjective well-being and stress on foreign language enjoyment: The mediating effect of positive language education

**DOI:** 10.3389/fpsyg.2023.1007534

**Published:** 2023-02-24

**Authors:** Anna Lia Proietti Ergün, Hande Ersöz Demirdağ

**Affiliations:** Department of Western Languages and Literatures, Translation and Interpreting, Yıldız Technical University, Istanbul, Türkiye

**Keywords:** positive language education, well-being, foreign language enjoyment, multilingualism, stress

## Abstract

The present study is the first to investigate the extent to which positive language education can improve foreign language enjoyment in the same learners. At the same time, it explores the relation between life quality variables, subjective well-being (SWB), and stresses that have emerged as important variables to explain foreign language enjoyment (FLE). Participants were 50 native speakers of Turkish, university students, 24 having a high proficiency in one language (English) and 26 having a high proficiency in two languages (French and English). Quantitative data were collected before and after the intervention (“3 Good Things” and “Use your Strength and Virtues in a Creative Way”) and qualitative data were collected at the end of the course. The results of pre-test and post-test analysis were not significant FLE (*t*(49) = −1.3, *p* > 0.05), SWB (*t*(49) = −0.58, *p* > 0.05), and stress (*t*(49) = −0.7, *p* > 0.05). Manova with a level of multilingualism as a fixed factor revealed that there was a significant difference in the pre-test and post-test *F*(3, 46) = 3.49, *p* < 0.023, Wilk’s Λ = 0.81, partial *η*^2^ = 0.023. The Between Subjects’ Test reveals a significant difference in the level of SWB (*F*(1, 48) = 7.04, *p* < 0.01) and FLE (*F*(1, 48) = 8.5, *p* < 0.005), but not for the level of stress (*F*(1, 48) = 0.29, *p* < 0.59). A subsequent set of regressions revealed that in pre-test there is not a significant effect of the factors on the dependent variable (*R*^2^ = 0.20, *R*^2^ adjusted = 0.140). The analysis after the intervention shows a significant effect of the SWB on FLE *R*^2^ = 0.35, *R*^2^ adjusted = 0.31, Beta = 0.25, *p* < 0.002. The analysis of the quantitative data supports the statistical analysis as students report that the intervention has helped to improve the atmosphere in the classroom as well as their general attitude in life and they have learned valuable knowledge about themselves as an added value to the acquisition of the new language. We conclude that positive language education can increase the level of subjective well-being in students and that, in turn, improves the level of positive emotions in the language classroom.

## 1. Introduction

In the last 10 years, driven by the progress in the field of positive psychology (PP; Seligman, 2002), research in foreign language acquisition has moved from being focused, or “obsessed” as described in Wang et al. (2021, p. 2), on the negative side of language learning (with special attention towards anxiety), to investigate positive emotions and the role they play in fostering language learning. Although the study of positive emotions in foreign language learning and teaching has started to blossom (Dewaele et al., 2019), the role of “outside the classroom” variables such as subjective well-being (SWB) or the level of perceived stress (PS) in the way the language lesson is experienced, is still barely investigated. Student well-being has increasingly become the focus of attention of scholars and policymakers so that, as stated in study of (Mercer, 2021, p. 20), “Learner well-being is a core ingredient of successful learning in the present and a curricula life skill goal for the future.” Mercer et al. (2018, p. 21) advocate that “learning a language can be thought of as a way in itself of enhancing well-being” and that the time is right for promoting positive language education: a teaching approach that does not focus only on the language but also includes activities that promote flourishing. This does not imply that language teachers must become a psychologist but rather that, as many teachers already do, adopting a teaching approach that promotes positive emotions and a sense of community in the classroom. The positive correlation between learning a foreign language and well-being has been assessed in previous studies (Oxford and Cuéllar, 2014; Proietti Ergün and Ersöz Demirdağ, 2022), but there is an ongoing discussion on the reason for this correlation. An explanation could be found in the broaden-and-build theory proposed by Fredrickson (2001, 2004), according to which positive emotions are not just markers of happiness and well-being. Positive emotions such as enjoyment expand students’ ability to process and acquire new information. Positive emotions experienced in the classroom may foster well-being which, in turn, helps to experience more positive emotions. Thanks to the instrument developed by Dewaele and MacIntyre (2014) and its shorter version developed and validated by Botes et al. (2021), there is the possibility of reliably measuring at least one of the positive emotions experienced in the foreign language classroom, Foreign Language Enjoyment (FLE) that “can be described as a broad, overarching positive emotional variable that is designed to encapsulate a positive disposition towards the FL learning process, towards peers, and towards teachers” (Botes et al., 2020b, p. 3–4).

Is it not yet clear to what extent Positive Psychology Interventions (PPI) adapted for the foreign language classroom can improve the level of positive emotions and if they really have an impact on the level of mental well-being and stress students experience in their life. At the moment, all we know is that PPI in the language classroom brought benefit in terms of well-being (Gregersen, 2016; Li and Xu, 2019), improving language skills (Piasecka, 2016; Leung et al., 2019; Hui et al., 2020; Abdolrezapour and Ghanbari, 2021), and diminishing Foreign Language Classroom Anxiety (Jin et al., 2021).

This study concentrates on FLE because, as stated before, it is a well-conceptualized, reliably measurable, positive emotion. It will explore how this classroom related emotion correlates with two variable that holistically measure subjective well-being and perceived stress. The novelty of this study is not restricted to the investigation of the relation between SWB, PS, and FLE, but it investigates also whether a positive language education approach (MacIntyre et al., 2019) along with positive psychology interventions may change the relations among these variables and to what extent individual differences may account for this variability. To this end, quantitative and qualitative data were collected from two classes of Turkish university students with different levels of multilingualism. Data were collected at the beginning of the semester and again after 14 weeks of positive language education and two specific positive psychology interventions (3 Good Things, Using Signature Strengths in a New Way; Seligman et al., 2009). We believe that this study, despite the limitations that we will discuss in the final section, may have important implications for the future direction of foreign language teaching.

## 2. Literature review

### 2.1. Well-being in positive psychology and education

“As an operational definition, SWB is most often interpreted to mean experiencing a high level of positive affect, a low level of negative affect, and a high degree of satisfaction with one’s life” (Deci and Ryan, 2008, p. 1). There is no universal agreement among researchers on what Well-being means (Huppert and Ruggieri, 2018). The debate of what is a good life goes back, in the Western World, to Ancient Greek philosophers. [The philosophic debate regarding the definition and composition of a good life can be traced all the way back to Ancient Greece.] According to Aristoteles, the good life was the one spent in the pursuit of higher ideals and virtues or Eudaimonia. On the other end of the spectrum, Epicurus claimed that the good life was reached through the satisfaction of needs or Hedonism. This ancient debate gained again a central role when, in 2000, the millennian issue of the influential journal “American Psychologist” was dedicated to the emerging science of Positive Psychology (PP), the difference between PP and all the previous philosophical debates on what is a good life, lies in the fact that rigorous scientific methods are applied to investigate what goes well in life and to evaluate the outcome of interventions (Seligman and Csikszentmihalyi, 2000).

Research, since the early onset of PP as a scientific field, pointed out the fact that only one component, be it Eudaimonia or Hedonism, is not sufficient to guarantee the achievement of a good life and that subjective well-being is a complex construct that depends on many different factors such as a sense of purpose, meaningful relations, and physical, psychological, and social health. These developments in research encouraged Seligman to propose his theory’s fundamental dimensions that constitute SWB, namely, Positive Emotion, Engagement, Relationships, Meaning, and Accomplishment, known by the acronym PERMA (Seligman, 2012, 2018).

The encouraging results obtained with PP based intervention in enhancing SWB, lead Seligman et al. (2009) to advocate that PP-based protocols should be implemented in schools, “… were it possible, well-being should be taught in school on three grounds: as an antidote to depression, as a vehicle for increasing life satisfaction, and as an aid to better learning and more creative thinking.” (Seligman et al., 2009, p. 295). Call of Seligman et al. (2009) did not go unanswered, and research has assessed that PP interventions targeting well-being improve academic achievement (e.g., Hughes and Kwok, 2007; Reyes et al., 2012).

### 2.2. SWB in second language acquisition

In the field of second language acquisition, student well-being has been conceptualized for the first time by Oxford (2016, 2018) who proposed the EMPATHIC model. She affirmed that the five core elements of well-being (Positive Emotion, Engagement, Relationships, Meaning, and Accomplishments) proposed by Seligman (2012); are not enough to define language students’ well-being. Therefore, Oxford (2016, 2018) extended model of Well-Being of Seligman (2012) to a new model that includes aspects relevant to SLA: “E: emotion and empathy; M: meaning and motivation; P: perseverance, including resilience; A: agency and autonomy; T: time; H: habits of mind; I: intelligence; C: character strengths; and S: self-factors, especially self-efficacy” (Oxford, 2018, p. 27). The study of well-being in foreign language students is still at an early stage. Oxford (2014) has been the first to focus on language student’s well-being. In her study she reported on two students that according to her were to be placed on opposite ends of the well-being spectrum and she found a correlation between the levels of well-being reported by the two learners with their ability to be strategic learners. Oxford and Cuéllar (2014) analyzed the narrative of five university students learning Chinese in Mexico using the PERMA framework. The students reported that learning a new language significantly contributed to their well-being. Well-being and attributional patterns were studied by Fatemi and Asghari (2016) in Iranian university students learning English; they found a positive correlation between their well-being and their attributional patterns in learning English. Finally, Chen and Zhang (2020) investigated the impact of well-being on EFL learners’ language performance finding a significant correlation between the two variables.

Few are the studies exploring the benefit of positive psychology intervention in the foreign language classroom. Gregersen (2016), for example, concentrates on PP activities regarding gratitude, altruism, music, pets, exercise, and laughter. During a period of 12 weeks, five students (three Brazilian and two Japanese) enrolled in a United States university in an English intensive program were paired with a partner to participate in conversational activities. Language partners were instructed to implement the PP activities. Data analysis showed how those interventions fostered resilience to keep on with an activity such as language learning, often perceived as frustrating, as well as improving the level of enjoyment in learning a new language. Piasecka (2016) concentrated on Character Strengths. She used poetry to enlighten students’ character strengths and although in the beginning students were not enthusiastic about the literature course, they later found the activities engaging, and the character strengths that they used in connection with poetry, such as creativity, courage, curiosity, open-mindedness, appreciation of beauty were found to be connected to self-efficacy and life satisfaction. Jin et al. (2021) tested the effect of relaxation and reminiscing exercises. Students of English as a foreign language in the experimental group were asked, after a moment of relaxation, to recall their progress in learning the new language. The experimental group and the control group were tested to assess their level of FLCA 30 day before the beginning of the experiment and 30 days after. Results showed that the level of anxiety was diminished significantly in the experimental group but was stable in the control group.

### 2.3. Stress and foreign language enjoyment

The word “stress” is often used interchangeably with the word anxiety. In psychology, it is conceptualized as the way an individual is coping in certain situations, especially those that seem to place a demand that exceeds the individual resources.

“Stress has a different meaning for different people under different conditions. A working definition of stress that fits many human situations is a condition in which an individual is aroused and made anxious by an uncontrollable aversive challenge” (Fink, 2016, p. 4). Stress triggers the fight or flight response (Cannon, 1929),^1^ which evolved as a survival mechanism to allow the individual to fight the danger or flight to a safe place. Unsurprisingly stress has been found negatively correlated with well-being (Zika and Chamberlain, 1987; Chatters, 1988; Suh et al., 1996) and PP interventions seem to decrease stress and increase happiness (King, 2001; Compton, 2005; Lyubomirsky et al., 2006).

On the opposite end of the “fight or flight” response, there is Broaden-and-Build Theory of Fredrickson (2001, 2003) according to which experiencing positive emotions, such as joy and contentment, trigger a physiological response that enables the individuals to broaden their interests and build new knowledge. Grounded in Broaden-and-Build Theory of Fredrickson (2001, 2003) and in Positive Psychology (Seligman and Csikszentmihalyi, 2000), Foreign Language Enjoyment (FLE), emerged as a construct for the first time in Dewaele and MacIntyre (2014).

Dewaele and MacIntyre (2016) described FLE as a complex emotion that is aroused “when people not only meet their needs but exceed them to accomplish something new or even unexpected” (p. 217). We acknowledge the recent reflections of Li and colleagues (Li and Xu, 2019; Li, 2020; Li and Dewaele, 2020; Dewaele, 2022) on grounding FLE in the control-value theory (Pekrun et al., 2006) which has a specific focus on education and particularly in the triggers and outcomes of emotions, but, as stated in Dewaele (2022, p. 193) “control-value theory does not consider the interaction between positive and negative emotions, something that is central in the broaden-and-build theory that emphasizes the essential role of positive emotions in neutralizing the after-effects of negative emotions.” Following reasoning of Dewaele (2022), this study uses Broaden-and-Build Theory of Fredrickson (2001, 2003) as a theoretical framework to investigate the relation among SWB, PS, and FLE. Moreover, it intends to reach a better understanding of to what extent this relation can be influenced by PP interventions adapted for the la foreign language classroom.

### 2.4. Research questions

In the light of this literature review, we can conclude that, although there is a recommendation of focus on learners’ well-being and a call to implement positive linguistic education (PLE; Mercer et al., 2018), the effects of PLE on students’ SWB, PS, and FLE are still largely undocumented. Although FLE is a well-researched positive emotion, there is still a need to investigate it in relation to more general variables that include students’ perceptions of their quality of life. This study will contribute to advancing our knowledge of the effect of PLE on FLE, SWB and PS, by answering three research questions:

*RQ 1:* Does positive language education (PLE) PP interventions change the level of SWB, PS, and FLE?

*RQ 2:* Does PLE and PP interventions change the way SWB and PS explain the level of FLE?

*RQ 3:* Does Degree of Multilingualism (DM) play a role in the transformation?

## 3. Method and materials

### 3.1. Participants

The sample consisted of *n* = 50 university students in Turkey. The average age of the sample was 21.9 years (SD = 3.3). Most participants were female (*n* = 39). Participants were learning Italian (*n* = 26) and French (*n* = 24) at A1 (beginners) level. Turkish is the native language of all the participants; the group learning French had a C1 level of English, and they were enrolled in an EFL teaching degree program. The group learning Italian had a B2 level in French and in English, and they were enrolled in an interpreting degree program (Table 1).

**Table 1:** Reliability statistics for the variables (Cronbach’s alpha).

	SWB	PS	FLE
*Variable (Pre-test)*			
Cronbach’s Alpha	0.895	0.655	0.858
Number of items	14	9	14
*Variable (Post-test)*			
Cronbach’s Alpha	0.895	0.655	0.858
Number of items	14	9	14

### 3.2. Materials

The study was conducted using an online survey tool (Google form). The questionnaire started with a demographic survey followed by three tools measures to assess Subjective Well-Being, Perceived Stress, and Foreign Language Enjoyment. The three full scales are presented in the Appendix section.

1. Subjective Well-Being (SWB): To assess levels of Well-Being, participants were asked to complete the Turkish version of the Warwick-Edinburgh Mental Well-Being Scale (SWEMWBS) validated in study of Keldal (2015). The SWEMWBS was developed to identify levels of Well-being and includes elements to assess both hedonic and eudaimonic aspects of positive mental health (Tennant et al., 2007).
  Permission to use this scale was granted by email by the copyright owner. The scale measures general levels of perceived well-being with 14 items posed as positive statements, i.e., “I have been feeling optimistic about the future.” Participants were asked to rate how often they agree with the statement on a 5-points Likert response scale, with 1 indicating “never” and 5 indicating “always.” Reliability statistics were satisfactory for the 14 items (see Table 1) in pre-test and post-test. Possible scores for this task range from 14 to 70, with higher scores indicating higher positive mental well-being.
2. Perceived stress: To measure participants’ perception of life situations as stressful, The Turkish validated version of the Perceived Stress Scale (PSS; Yerlikaya and İnanç, 2007) was used. The scale is composed of 14 items; participants rate how often they experience a certain feeling and thought. Items were posed as questions, i.e., “In the last months, how often have you been upset because of something that happened unexpectedly?” Participants must respond on a 5-point Likert response scale, with 1 indicating “never” and 5 “very often.” In this task, scores ranged from 14 to 70, with higher scores associated with higher levels of stress. Reliability statistics were satisfactory (Dörnyei and Taguchi, 2010; see Table 1).
3. Foreign Language Enjoyment (FLE): To measure Foreign Language Enjoyment, participants were given a translated version of the Short Form of the Foreign Language Enjoyment Scale (S-FLES) developed by Botes et al. (2021), based on the original 21 items scale used in Dewaele and MacIntyre (2014). The questionnaire was translated by a professional native Turkish translator and reviewed by two university professors. Participants were asked to rate to what extent they agreed with the statements in the questionnaire on a 5-point Likert scale, with 1 indicating “strongly disagree” and 5 indicating “strongly agree.” All items were positively phrased and included sentences such as “I enjoy it,” and “I do not get bored.” Internal consistency was satisfactory (see Table 1) for pre-test and post-test and students revealed high levels of FLE (scores ranged from a minimum of 9 to a maximum of 45).

### 3.3. Character strengths and virtues

As the result of a deep theoretical study of religious doctrines, ancient and recent philosophy, and cultural tradition, Peterson and Seligman (2004) were able to describe six “core virtues.” Theoretically, courage, justice, humanity, temperance, transcendence, and wisdom are common virtues in all cultures and philosophical/religious approaches. They subsequently identified 24 “character strengths” that could be reconducted to those six virtues. Character strengths are universally valued character traits that can contribute to a meaningful life (Peterson and Seligman, 2004). To help people to identify their character strengths, they created an online survey tool.^2^

### 3.4. Procedure

The two teachers involved in this study selected two classes of absolute beginners in French and Italian. The two teachers agreed on a common approach for teaching, which was based on the affective-humanistic approach advocated by Balboni (2012). Every class was always starting with the teacher asking every student about what was going well in their lives (instead of a generic “how are you”) or with a few moments of music. On week 9 of the course, they were asked to do “Silver lining” exercise of Seligman et al. (2009). They were asked to keep a diary for 1 week where they had to write down simple sentences in the target language concerning three good things that had happened in their life and how that made them feel. The following week (week 10), the teachers proposed another intervention based on the character strengths inventory the “signature strength intervention” (Seligman et al., 2005, 2009), they were asked to access the *VIA* on-line survey tool to identify their signature strengths and then use them in a creative way every day for 1 week and report the event and how it made them feel in their journal. The teachers controlled the diaries daily and included in the study only those subjects that had participated in both the interventions.

## 4. Data collection and analysis

The data were collected two times *via* an online questionnaire. The convenience sampling technique (Dörnyei, 2007) was employed to collect data for this study. Before the courses started, researchers asked students to fill the SWEMWBS, the PSS and the FLE in Turkish online, after the completion of the course students filled again the same scales and were asked to answer three open-end questions. In this phase, it was decided to collect quantitative and qualitative data together, because it was less time-consuming for the students and very efficient. Only the subjects that had participated in pre-test, in post-test, and had taken the two PP interventions were included in the study.

**Figure 1:**
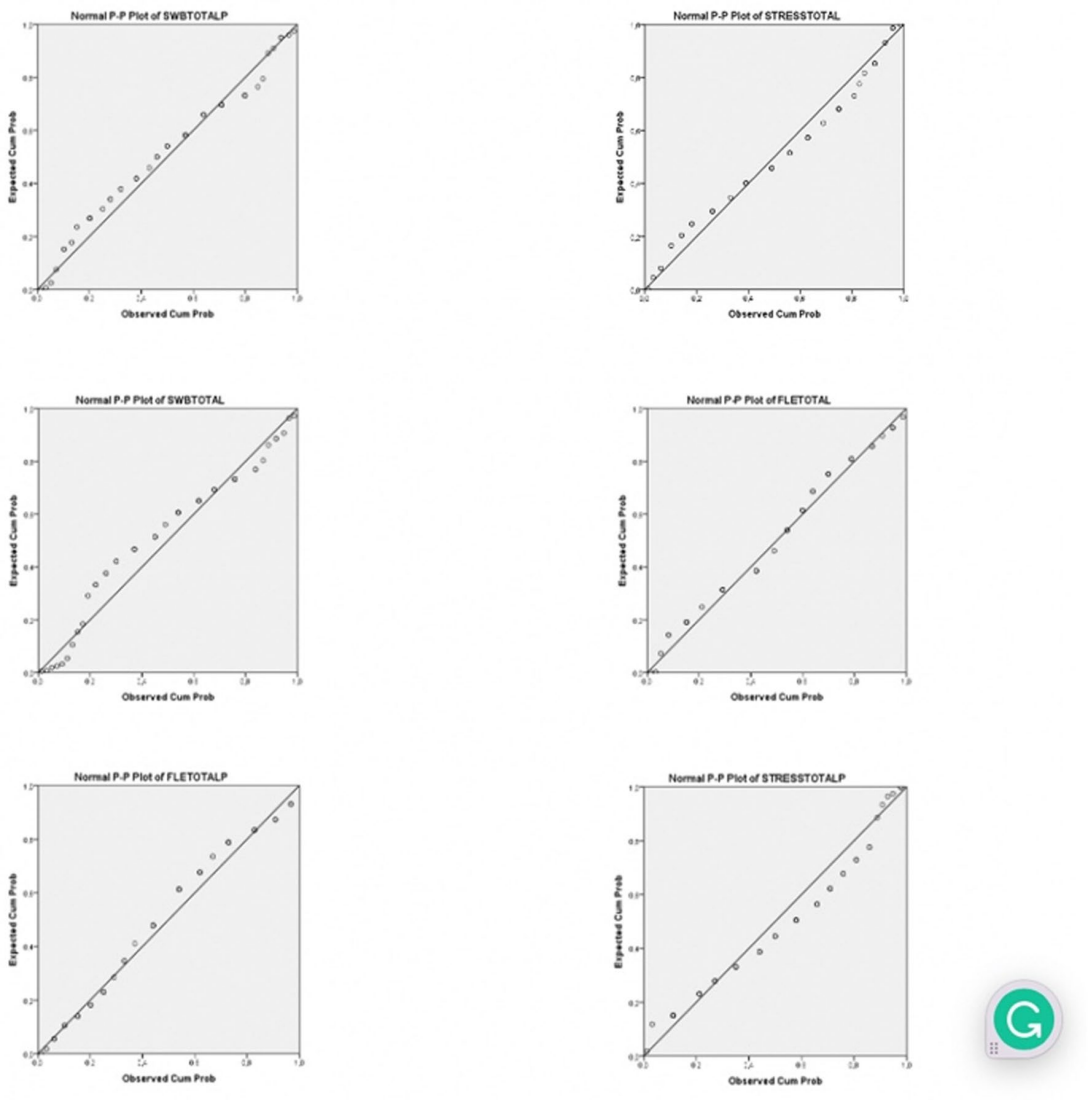
P-P plots for normal distribution.

Data were analyzed in different phases using the Statistical Package for the Social Sciences (SPSS 23). The three study variables (FLE, WB, and PS) were tested for internal consistency. To test internal consistency, Conroy (2018) suggests a minimum of 30 samples. As the internal consistency of the tools employed in this study has already been validated in a large number of studies, the small sample size (50 subjects) can be considered sufficient to conduct Cronbach’s alpha test internal consistency (Table 1), The internal reliability for PSS was a bit low end (Cortina, 1993), but given that the internal validity of the scale had been controlled and validated for consistency in multiple studies (i.e., Yerlikaya and İnanç, 2007; Örücü and Demir, 2009), it was concluded that internal consistency could be considered satisfactory. Kolmogorov–Smirnov tests with Lilliefors Significance Correction were performed, and the FLE distribution was found to be normal (KS = 0.094, *p* > 0.05). The P-P plots (Figure 1) indicate that the data collected for the variables in pre-test and post-test follow a normal distribution. Multiple *t*-tests and Manova analysis were used to investigate the research questions, followed by multiple regression analysis (enter method). Bootstrapped statistics were used to avoid Type I and Type II errors caused by the small sample size (Plonsky and Oswald, 2014). Qualitative data were analyzed using thematic analysis, extracting the core concepts indicated by the students. In the discussion section, quantitative and qualitative data are elaborated together.

The first research question was to understand whether positive education along with the classical positive psychology intervention would have created a difference among one or more of the variables; to this end, a paired sample *t*-test with bootstrap (Table 1) was conducted to compare pre-test and post-test conditions. No significant difference was found for FLE in pre-test condition (*M* = 35.5, SD = 5.14) and post-test conditions (*M* = −36.3, SD = 5.14); SWB in pre-test condition (*M* = 50.7, SD = 8.5) and post-test conditions (*M* = 50, SD = 9.72); STRESS in pre-test condition (*M* = 48.7, SD = 6.93) and post-test conditions (*M* = 48.9, SD = 6.69).

To answer the second research question of whether PLE and PP interventions change the way SWB, and PS explain the level of FLE, a Multiple regression analysis (enter method) was used. Values for the variance inflation factor (VIF), which quantifies the severity of multicollinearity, were around 1; Kutner et al. (2004) suggest a value higher than five implies multicollinearity; consequently, the data in this study are assumed to be risk-free. Regression analysis revealed that in the pre-test there is not a significant effect of the factors on the dependent variable (*R*^2^ = 0.20, *R*^2^ adjusted = 0.140). The analysis after the intervention shows a significant effect of the SWB on FLE *R*^2^ = 0.35, *R*^2^ adjusted = 0.31, Beta = 0.25, *p* < 0.002 According to Plonsky and Ghanbar (2018), this result must be considered a small to medium effect size.

To answer the third research question, Multivariate Analysis of Variance (Manova) was used to determine the difference between the two groups regarding the change in levels, with a level of multilingualism as a fixed factor. The number size for this Manova was calculated using G*Power (Faul et al., 2007), and assessed to be 54 for an effect size *F* of 0.025, although our sample size fell short of this number, we assumed that the sample size criteria were still met. Values for the variance inflation factor (VIF), to assess the danger of multicollinearity, were around 1; Kutner et al. (2004) suggest a value higher than five implies multicollinearity; so we assumed that also the non-multicollinearity assumption was met so that the Manova could be run and revealed that there was a significant difference in the pre-test and post-test *F*(3, 46) = 3.49, *p* < 0.023, Wilk’s Λ = 0.81, partial *η*^2^ = 0.023. The Between Subjects’ Test reveals a significant difference in the level of SWB (*F*(1, 48) = 7.04, *p* < 0.01) and FLE (*F*(1, 48) = 8.5, *p* < 0.005), but not for the level of stress (*F*(1, 48) = 0.29, *p* < 0.59).

## 5. Students’ assessment of the course approach and PP interventions

Learners’ opinions about the course and the PP intervention, collected through three open questions, were very helpful in elucidating the quantitative results. After carefully reading the content of the collected answers, the themes were elaborated. Many answers contained multiple topics, so repeated coding was used. Each section will report the question and the data analysis.

### 5.1. What did you enjoy in this course?

**Figure 2:**
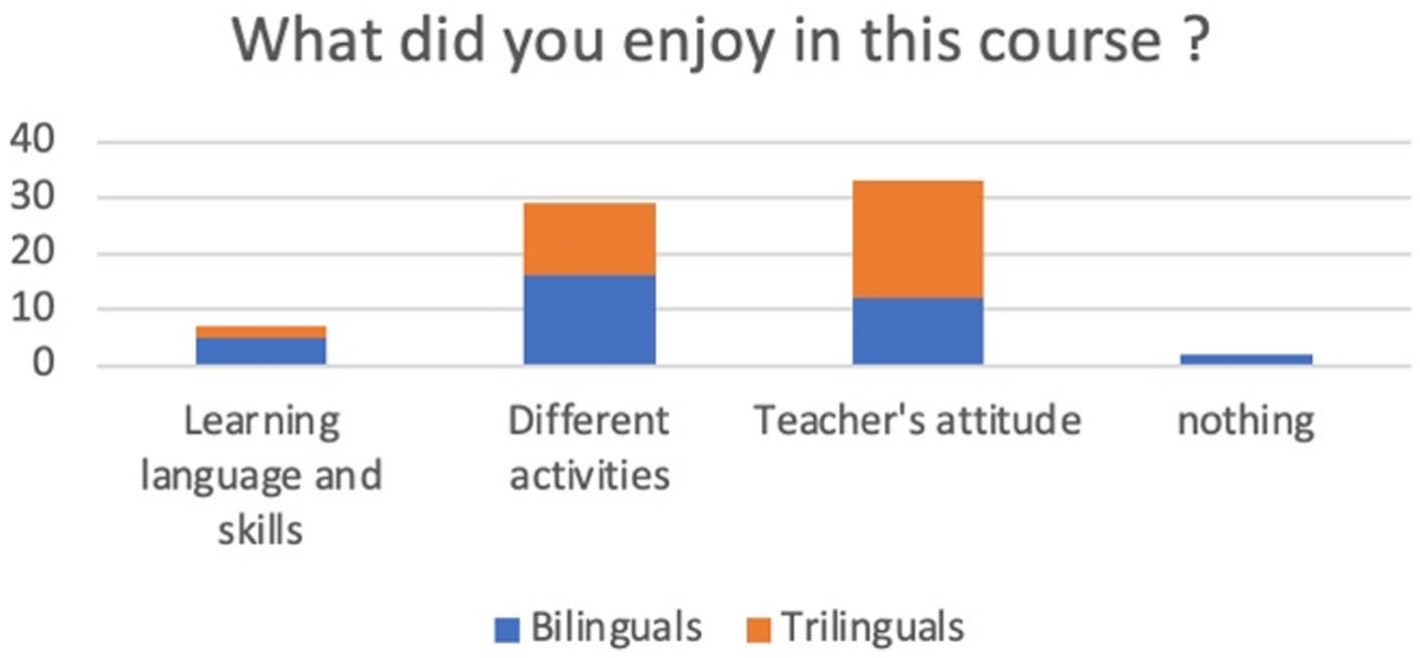
Histogram the answers to question 1.

After carefully reading the content of the collected answers, we classified the data into three categories: Many answers contained multiple topics, so we use repeated coding. The results of data coding are summarized in Table 2 and illustrated in Figure 2.

**Table 2:** Bootstrap results based on 1,000 bootstrap samples.

	Mean	Bootstrap				
		Bias	Std. Error	Sig. (2-tailed)	95% confidence interval	
					Lower	Upper
FLETOTAL – FLETOTALP	−0.82	−0.0066	0.5796	0.175	−1.92	0.37949
SWBTOTAL – SWBTOTALP	0.7	0.01536	1.20708	0.56	−1.63949	3.16
STRESSTOTAL – STRESSTOTALP	−0.18	0.01158	0.98243	0.854	−2	1.76

The teacher’s attitude stands out in the data as what the students’ appreciated the most.

The behavior of the teacher, or rather the conversations with the teacher have been flagged as a source of joy, for example, Student (S.) 26 affirms: “*I was happy to see our teacher*”; another student, S.22: “*I was pleased with our teacher’s encouraging, warm and sincere behavior in the class.”* S.11: “*I was pleased with our teacher’s encouraging, warm and sincere behavior in the class, The homework was practical and pleasurable.*”; S.38: “*we were talking with our teacher, and I appreciate her positive attitude.”*

The variety of activities proposed were the second thing that students appreciated most. Even though the questions were posed in the native language of the student, a student tried to answer in French: S.10: “*Écouter de la chanson française et apprendre de nouveaux mots*.” (“to listen to French songs and learn new words”) highlights how the student enjoyed learning new things and the fact that she feels comfortable using the foreign language even though the student is still a beginner. Students also find it natural to compare these language courses with others, for example, S.36 affirmed: “*if compared with other language courses this one was more active*.”

Only one student out of 50 declared that there was nothing she/he enjoyed in this course. So, we concluded that students appreciated this new approach and that they felt that the teacher was making a difference.

### 5.2. Themes emerged as an answer to question 2

**Figure 3:**
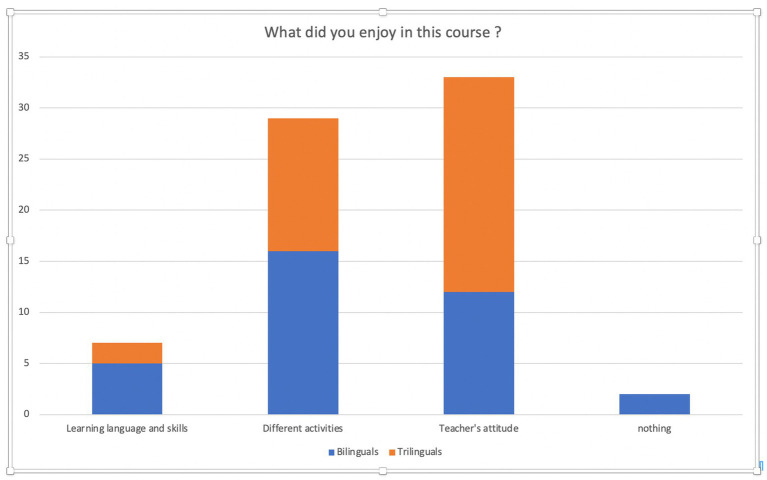
Histogram the answers to question 2.

When examining the results of this section, it appears that “happy” ranks first among both bilingual and trilingual students, followed by “willing to learn” (Table 3; Figure 3).

**Table 3:** Themes emerged as an answer to question 1.

	Learning language and skills	Different activities	Teacher’s attitude	Nothing
Bilinguals	5	16	12	2
Trilinguals	2	13	21	0

The answers were mostly referring to positive feelings and only two people (%4 of the total participants) answered that they were feeling stressed. Feeling good, happy, and willing to learn were the most frequent emotions the students experienced.

Willing to learn and feeling good or happy was expressed, by the students, as having been “enthusiastic *to learn more and I was especially happy as my reading comprehension improved*” (S.5) or as having “*a good time because I learned new things*” (S.19).

But we believe that the brief sentence of S.42 “*Even if it was for a short period, in the classroom I forgot the depressive environment*” bestows a special meaning to the work done by the teachers.

### 5.3. The two PP activities we did in the class made me feel

**Figure 4:**
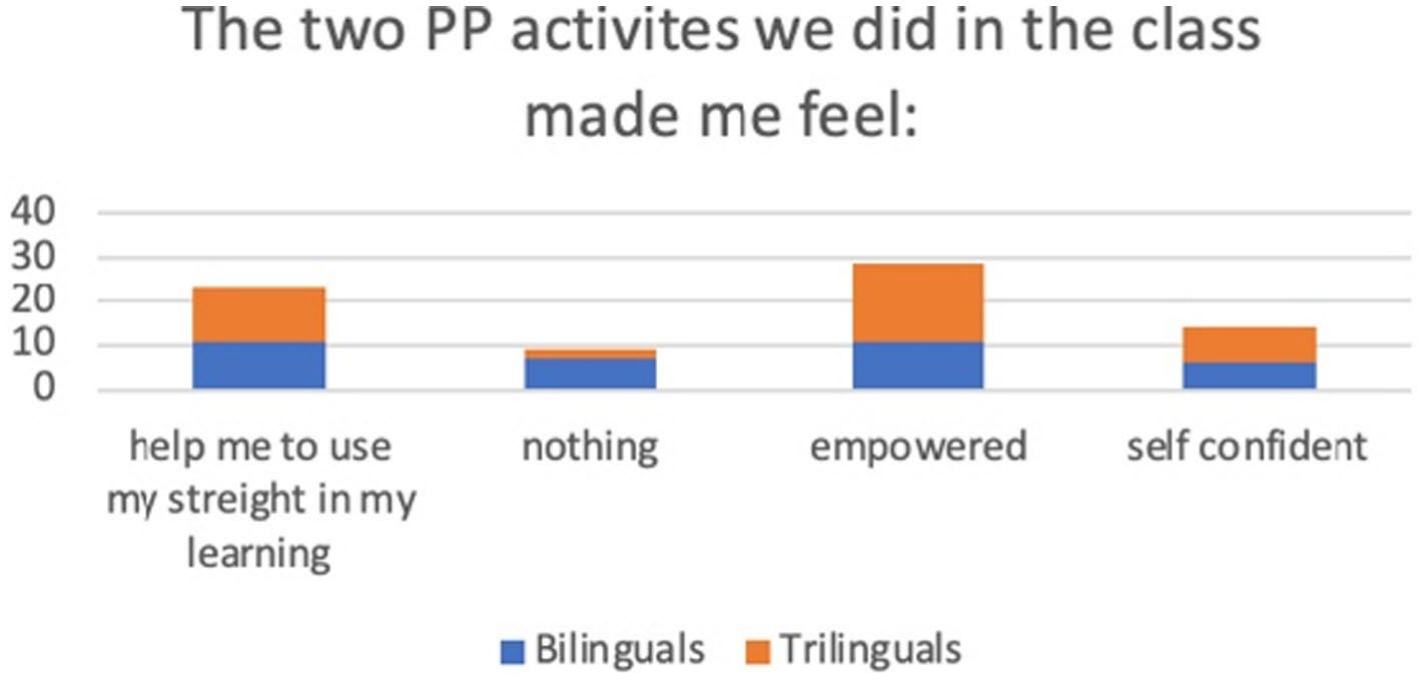
Histogram the answers to question 3.

It appears that the PP activities empowered students during language lessons. In addition, it seems to help in using their strengths in learning, rather than focusing on weaknesses, and they provide self-confidence. Yet there are many bilinguals (7, %30 of the total) that declare that PP activities have no influence in the way they feel (Table 4, Figure 4).

**Table 4:** Themes emerged as an answer to question 2.

	Willing to learn	Good	Happy	Stressed
Bilinguals	5	6	13	1
Trilinguals	8	2	15	1

The first PP intervention, the “three good things activity” helped students to feel a connection between the class and their life in general:

*S.23:* We write sentences about ourselves and what it goes well every day was nice and motivating.

*S.41:* The fact that we were asked to write down positive things in our lives every day in Italian class added a little more positive perspective.

*S.12:* We wrote down the things we did every day that made us feel good. The following week, we wrote about our strengths and how they reflect on our lives. Writing sentences in Italian every day allowed me to retain and better consolidate words in my mind.

The “signature strength” intervention was particularly appreciated, and it leads students to feel especially empowered, and more confident, and they take more pleasure in learning (Table 5).

**Table 5:** Themes emerged as an answer to question 3.

	Help me to use my strength in my learning	Nothing	Empowered	Self confident
Bilinguals	11	7	11	6
Trilinguals	12	2	18	8

*S1:* My strengths are perseverance, curiosity, and creativity. I realized that even though I had a hard time learning the language, I do not give up. And I’m curious about the words I do not know, I want to know their meaning.

*S35:* My sense of courage supported my participation in the lesson.

In conclusion, we can say that most students considered these exercises to empower them as language learners.

## 6. Discussion

The first aim of this study was to prove empirically the need for answering the call for positive language education (Mercer et al., 2018); in other words, we wanted to understand whether and to what extent positive language education and specific activities could influence the variable under observation. The *T*-tests show no statistically significant change in the level of PWB, PS, and FLE before and after the implementation of the course. Based on Broaden-and-Build theory, we would expect the level of SWB and FLE to increase and PS to decrease; in this regard, the results were disappointing. All the students started with a high level of SWB and FLE and moderate stress, and apparently, the course did not impact the level of the variables. Yet, if we look at the standard deviations, we can observe that they increase for SWB and FLE, but it remains the same for PS, suggesting that some students benefit more from this course than others. The quantitative data, on the other hand, paints a picture of students that have enjoyed the positive education approach as well as the interventions and that they feel empowered also on their ability to learn better.

The set of regressions run to investigate about the relation between SWB, PS, and FLE at the beginning of the courses and at the end of the course show us that before the intervention, SWB is not related to FLE but with the progress of the course SWB became a predictor for FLE and the two variables became interrelated. The change in the relationship between SWB and FLE is meaningful and supported by the qualitative data analyses. The students became conscient of the link between what is happening in their life and what is happening in the class. As they state, they find learning fun and meaningful. Qualitative data also inform us that the teacher, not unexpectedly, is central to this process. Dewaele and MacIntyre (2022) had already researched to what extent the teacher is central to FLE and that teacher friendliness and encouraging approach are correlated to a higher level of FLE (Li et al., 2018: Dewaele et al., 2019; Dewaele and MacIntyre, 2022). What is new in this study, and it is worth pointing out, is that the approach of the teachers is not merely based on their goodwill but on the strategic and planned decision to try a new approach (positive language education) and to implement PP interventions. Teachers were aware of what they were doing and there was a conscious effort to make language teaching a tool to improve students’ SWB (Mercer et al., 2018) and investigate if this would have an impact on FLE. Qualitative data complete the picture given by the quantitative study providing additional information on the factors that mediated the relation between well-being and FLE, that is that they felt generally happier, they understood how to use their strength to become better learners, and felt inspired by the course, confirming previous studies implementing PP interventions (Gregersen, 2016; Piasecka, 2016). PS was constant in pre-test and post-test and its relationship with the other variable did not change, this would suggest that stress, as anxiety, is an independent variable from SWB and FLE (Dewaele and MacIntyre, 2014).

A more fine-grained analysis of the data was conducted to understand whether the degree of multilingualism (DM) could explain some of the variability in the data. Based on previous studies on the relationship between multilingualism and FLE (Dewaele and MacIntyre, 2014; Dewaele et al., 2019; Botes et al., 2020a) DM, SWB, PS, and FLE (Proietti Ergün and Ersöz Demirdağ, 2022), the hypothesis was that DM could have a significant impact on the variable. To this end, a Manova was conducted revealing that students learning their fourth language had a significantly higher level of SWB and FLE although no statistical significance was obtained for PS. This confirmed the initial doubt about some students benefiting more than others from the course. The reason why DM may have an influence on the level of FLE has been discussed and amidst the theories is that “multilingual may enjoy learning new languages more because they have more experience with them and may have developed clear strategies for language learning” (Dewaele, 2022, p. 195), SWB enters the equation confirming students’ self-reports about how learning a new language contributes to their general well-being (Oxford and Cuéllar, 2014).

The figures in this study are encouraging and qualitative data confirm that positive language education and PPI helps students to become not only happier but also better learners. Moreover, we believe that S.42’s statement “Even if it was for a short period, in the classroom I forgot the depressive environment,” by itself is enough to claim that the call for Positive Language Education (Mercer et al., 2018) should not go unattended.

This study, with the limitations that will be discussed ahead, shed a light on the importance of taking care of students’ well-being in the language classroom. We feel that the results of this research have serious implications for in-service language teachers as well as for foreign language teaching programs. We believe that positive language education should be part of the training of every teacher, and foreign language coursebook design should include PP-based activities.

This study presents some limitations. First, the research sample was small, yet this limitation has been mitigated by using bootstrap statistics and collecting quantitative data. Secondly, the students were from the same country and from the same university, which limited diversity. We believe that there is a need for expanding this kind of research to larger and more diverse groups of learners. Another limitation concerns the qualitative data that were collected just by posing three questions online, future studies can make use of other instruments such as classroom observations or focus groups so that the relation between SWB and FLE could be better understood.

## 7. Conclusion

This study aimed to contribute to the understanding of the relationship between positive affect outside the class (SWB and PS) and inside the class FLE; furthermore, it focused on the mediating role that PLE and PP interventions could have on the variable in object. Results were encouraging, we assessed that after 14 weeks of course implementing PLE and positive intervention, SWB started to have a moderate effect on FLE. Students’ answers to open questions confirmed that teachers’ positive attitudes and knowing their strengths inspired and empowered them. The DM is confirmed to be a very important variable in foreign language learning, students with a higher DM benefitted more from the course regarding SWB and FLE.

This study takes up the call for a PLE (Mercer et al., 2018) and shows the need for investigation on the relationship between SWB and foreign language learning. The present study has implications for pre-service and in-service teachers, and also, as proposed by (Mercer, 2021), for Foreign Language Teaching program at the university. PLE should be explored in all its aspects to promote a language teaching approach that promotes the well-being of the students. The teachers should be empowered with FLE, as they are one of the primary sources of happiness for their students. Finally, teachers should be supported by schools that should see students’ well-being as important as any other academic goal.

## Data availability statement

The raw data supporting the conclusions of this article will be made available by the authors, without undue reservation.

## Ethics statement

Ethical review and approval was not required for the study on human participants in accordance with the local legislation and institutional requirements. The patients/participants provided their written informed consent to participate in this study.

## Author contributions

All authors listed have made a substantial, direct, and intellectual contribution to the work, and approved it for publication.

## Conflict of interest

The authors declare that the research was conducted in the absence of any commercial or financial relationships that could be construed as a potential conflict of interest.

## Publisher’s note

All claims expressed in this article are solely those of the authors and do not necessarily represent those of their affiliated organizations, or those of the publisher, the editors and the reviewers. Any product that may be evaluated in this article, or claim that may be made by its manufacturer, is not guaranteed or endorsed by the publisher.

## Footnotes

^1^The fight or flight response (Cannon, 1929) is still widely accepted but there is an on-going discussion on the need to improve the model (i.e., Bracha et al., 2004).

^2^ https://www.viacharacter.org

## Appendix

**SWEMWBS scale**

Below are some statements about feelings and thoughts.

Please mark the number that best describes your experience of each over the last 2 weeks

(1= None of the time, 2= Rarely, 3= Some of the time, 4 = often, 5= All of the time)

I’ve been feeling optimistic about the future

I’ve been feeling useful

I’ve been feeling relaxed

I’ve been dealing with problems well

I’ve been thinking clearly

I’ve been feeling close to other people

I’ve been able to make up my own mind about things

**FLE scale**

To what extent do you agree with the following statements? (1 = Strongly disagree, 2 = Disagree, 3 = Undecided, 4 =

Agree, 5 = Strongly agree)

1. The teacher is encouraging

2. The teacher is friendly

3. The teacher is supportive

4. I enjoy it

5. I’ve learned interesting things

6. I am proud of my accomplishments

7. We form a tight group

8. We laugh a lot

9. We have common ‘legends,’ such as running jokes

**The Perceived Stress Scale (PSS)**

For each question choose from the following alternatives:

(1 = never, 2 =almost never, 3 = sometimes, 4 = fairly often, 5 = very often)

1. In the last month, how often have you been upset because of something that happened unexpectedly?

2. In the last month, how often have you felt that you were unable to control important things in your life?

3. In the last month, how often have you felt nervous and ‘stressed’?

4. In the last month, how often have you dealt successfully with irritating life hassles?

5. In the last month, how often have you felt that you were e#ectively coping with important changes that were occurring in your life?

6. In the last month, how often have you felt con!dent about your ability to handle your personal problems?

7. In the last month, how often have you felt that things were going your way?

8. In the last month, how often have you found that you could not cope with all the things that you had to do?

9. In the last month, how often have you been able to control irritations in your life?

10. In the last month, how often have you felt that you were on top of things?

11. In the last month, how often have you been angered because of things that happened that were outside of your control?

12. In the last month, how often have you found yourself thinking about things that you have to accomplish?

13. In the last month, how often have you been able to control the way you spend your time?

14. In the last month, how often have you felt difficulties were piling up so high that you could not overcome them

## References

[ref2] Abdolrezapour P. Ghanbari N. (2021). The effect of positive psychology intervention on EFL learners’ listening comprehension. J. Psycholinguist. Res. 50, 1159–1180. doi: 10.1007/s10936-021-09780-5, PMID: 33909199

[ref7] Proietti Ergün A. L. Ersöz Demirdağ H. (2022). The relation between Foreign Language Enjoyment, subjective wellbeing, and perceived stress in multilingual students. Journal of Multilingual and Multicultural Development. 1–13.

[ref8] Balboni P. E. (2012). Le sfide di Babele. Torino: Insegnare le lingue nelle società complesse, Utet Libreria.

[ref10] Botes E. Dewaele J.-M. Greiff S. (2020a). The power to improve: effects of multilingualism and perceived proficiency on enjoyment and anxiety in foreign language learning. Eur. J. Appl. Linguist. 8, 279–306. doi: 10.1515/eujal-2020-0003

[ref11] Botes E. Dewaele J.-M. Greiff S. (2020b). The foreign language classroom anxiety scale and academic achievement: an overview of the prevailing literature and a meta-analysis. J. Psychol. Lang. Learn. 2, 26–56. doi: 10.52598/jpll/2/1/3

[ref12] Botes E. Dewaele J. Greiff S. (2021). The development and validation of the short form of the foreign language enjoyment scale. Mod. Lang. J. 105, 858–876. doi: 10.1111/modl.12741

[ref14] Bracha H. S. Ralston T. C. Matsukawa J. M. Williams A. E. Bracha A. S. (2004). Does "fight or flight" need updating? Psychosomatics 45, 448–449. doi: 10.1176/appi.psy.45.5.448 15345792

[ref15] Cannon W. B. (1929). Bodily Changes in Pain, Hunger, Fear and Rage: An Account of Recent Research into the Function of Emotional Excitement. 2nd Edn. New York: Appleton-Century-Crofts.

[ref17] Chatters L. M. (1988). Subjective well-being evaluations among older black Americans. Psychol. Aging 3, 184–190. doi: 10.1037/0882-7974.3.2.184 3268258

[ref18] Chen Z. Zhang P. (2020). Trait emotional intelligence and second language performance: a case study of Chinese EFL learners. J. Multiling. Multicult. Dev. 43, 731–745. doi: 10.1080/01434632.2020.1767633

[ref20] Compton W. C. (2005). Introduction to Positive Psychology. Belmont, CA: Thomson Wadsworth.

[ref21] Conroy R.. (2018). *The RCSI Sample Size Handbook*.

[ref22] Cortina J. M. (1993). What is coefficient alpha? An examination of theory and applications. J. Appl. Psychol. 78, 98–104. doi: 10.1037/0021-9010.78.1.98

[ref25] Deci E. L. Ryan R. M. (2008). Hedonia, eudaimonia, and well-being: an introduction. J. Happiness Stud 9, 1–11. doi: 10.1007/s10902-006-9018-1

[ref26] Dewaele J.-M. (2022). “Enjoyment” in The Routledge Handbook of Second Language Acquisition and Individual Differences. eds. Li S. Hiver P. Papi M. (London: Routledge), 190–206.

[ref28] Dewaele J.-M. Chen X. Padilla A. M. Lake J. (2019). The flowering of positive psychology in foreign language teaching and acquisition research. Front. Psychol. 10:2128. doi: 10.3389/fpsyg.2019.02128, PMID: 31607981 PMC6769100

[ref31] Dewaele J.-M. MacIntyre P. D. (2014). The two faces of Janus? Anxiety and enjoyment in the foreign language classroom. Stud. Second Lang. Learn. Teach. 4, 237–274. doi: 10.14746/ssllt.2014.4.2.5

[ref32] Dewaele J.-M. MacIntyre P. D. (2016). “Foreign language enjoyment and foreign language classroom anxiety. The right and left feet of the language learner” in Positive Psychology in SLA. eds. MacIntyre P. D. Gregersen T. Mercer S. (Bristol: Multilingual Matters), 215–236.

[ref33] Dewaele J.-M. MacIntyre P. D. (2022). Do flow, enjoyment and anxiety emerge equally in English foreign language classrooms as in other foreign language classrooms? Rev. Bras. Linguíst. Apl. 22, 156–180. doi: 10.1590/1984-6398202218487

[ref35] Dörnyei Z. (2007). Research Methods in Applied Linguistics. Oxford: Oxford University Press.

[ref36] Dörnyei Z. Taguchi T. (2010). Questionnaires in Second Language Research: Construction, Administration, and Processing. 2nd Edn.. New York, NY: Routledge.

[ref37] Fatemi A. H. Asghari A. (2016). The role of psychological well-being on university EFL learners’ attributional patterns. J. Educ. Soc. Res. 6, 189–197. doi: 10.5901/jesr.2016.v6n1p189

[ref38] Faul F. Erdfelder E. Lang A.-G. Buchner A. (2007). G*power 3: a flexible statistical power analysis program for the social, behavioral, and biomedical sciences. Behav. Res. Methods 39, 175–191. doi: 10.3758/BF03193146, PMID: 17695343

[ref39] Fink G. (2016). “Stress, definitions, mechanisms, and effects outlined: lessons from anxiety” in Stress Concepts, Cognition, Emotion, and Behavior. ed. Fink G. (Cambridge, MA: Elsevier Academic Press), 3–11.

[ref40] Fredrickson B. L. (2001). The role of positive emotions in positive psychology: the broaden-and-build theory of positive emotions. Am. Psychol. 56, 218–226. doi: 10.1037/0003-066X.56.3.218 11315248 PMC3122271

[ref42] Fredrickson B. (2003). The value of positive emotions. Am. Sci. 91, 330–335. doi: 10.1511/2003.26.330

[ref43] Fredrickson B. L. (2004). The broaden-and-build theory of positive emotions. Philos. Trans. R. Soc. Lond. Ser. B Biol. Sci. 359, 1367–1378. doi: 10.1098/rstb.2004.1512, PMID: 15347528 PMC1693418

[ref45] Gregersen T. (2016). “The positive broadening power of a focus on well-being in the language classroom” in Positive psychology Perspectives on Foreign Language Learning and Teaching. eds. Gabryś-Barker D. Gałajda D. (Switzerland: Springer), 59–73.

[ref49] Hughes J. Kwok O. (2007). Influence of student–teacher and parent–teacher relationships on lower achieving readers’ engagement and achievement in the primary grades. J. Educ. Psychol. 99, 39–51. doi: 10.1037/0022-0663.99.1.39, PMID: 18084625 PMC2140005

[ref50] Hui A. N. Chow B. W. Y. Chan E. S. Leung M. T. (2020). Reading picture books with elements of positive psychology for enhancing the learning of English as a second language in young children. Front. Psychol. 10:2899. doi: 10.3389/fpsyg.2019.02899, PMID: 32038350 PMC6990431

[ref51] Huppert F. A. Ruggieri K. (2018). “Policy challenges: well-being as a priority in public mental health” in Oxford Textbook of Public Mental Health, Oxford Textbook. eds. Bhugra D.. (Oxford: Oxford Academic)

[ref53] Jin Y. Dewaele J.-M. Macintyre P. (2021). Reducing anxiety in the foreign language classroom: a positive psychology approach. System 101:102604. doi: 10.1016/j.system.2021.102604

[ref54] Keldal G. (2015). Warwick-Edinburgh mental iyi oluş ölçeği’nin Türkçe formu: Geçerlik ve güvenirlik çalışması. J. Happiness Well-Being 3, 103–115.

[ref56] King L. A. (2001). The health benefits of writing about life goals. Personal. Soc. Psychol. Bull. 27, 798–807. doi: 10.1177/0146167201277003

[ref57] Kutner M. H. Nachtsheim C. J. Neter J. (2004). Applied Linear Statistical Models. 4th Edn. New York: McGraw-Hill.

[ref58] Leung C. Y. Mikami H. Yoshikawa L. (2019). Positive psychology broadens readers’ attentional scope during L2 reading: evidence from eye movements. Front. Psychol. 10:2245. doi: 10.3389/fpsyg.2019.02245, PMID: 31636588 PMC6788394

[ref59] Li C. (2020). A positive psychology perspective on Chinese EFL students’ trait emotional intelligence, foreign language enjoyment and EFL learning achievement. J. Multiling. Multicult. Dev. 41, 246–263. doi: 10.1080/01434632.2019.1614187

[ref60] Li C. Dewaele J.-M. (2020). The predictive effects of trait emotional intelligence and online learning achievement perceptions on foreign language class boredom among Chinese university students. For. Lang. Teach. 5, 33–44.

[ref62] Li C. Jiang G. Dewaele J. M. (2018). Understanding Chinese high school students' foreign language enjoyment: validation of the Chinese version of the foreign language enjoyment scale. System 76, 183–196. doi: 10.1016/j.system.2018.06.004

[ref63] Li C. Xu J. (2019). Trait emotional intelligence and classroom emotions: a positive psychology investigation and intervention among Chinese EFL learners. Front. Psychol. 10:2453. doi: 10.3389/fpsyg.2019.02453, PMID: 31736840 PMC6834770

[ref64] Lyubomirsky S. Sousa L. Dickerhoof R. (2006). The costs and benefits of writing, talking, and thinking about life’s triumphs and defeats. J. Pers. Soc. Psychol. 90, 692–708. doi: 10.1037/0022-3514.90.4.692, PMID: 16649864

[ref66] MacIntyre P. D. Gregersen T. Mercer S. (2019). Setting an agenda for positive psychology in SLA: theory, practice, and research. Mod. Lang. J. 103, 262–274. doi: 10.1111/modl.12544

[ref67] Mercer S. (2021). An agenda for well-being in ELT: an ecological perspective. ELT J. 75, 14–21. doi: 10.1093/elt/ccaa062

[ref68] Mercer S. MacIntyre P. Gregersen T. Talbot K. (2018). Positive language education: combining positive education and language education. Theory Pract. Second Lang. Acquis. 4, 11–31.

[ref69] Örücü M. Ç. Demir A. (2009). Psychometric evaluation of perceived stress scale for Turkish university students. Stress. Health 25, 103–109. doi: 10.1002/smi.1218

[ref70] Oxford R. L. (2014). What we can learn about strategies, language learning, and life from two extreme cases: the role of well-being theory. Stud. Second Lang. Learn. Teach. 4:593. doi: 10.14746/ssllt.2014.4.4.2

[ref71] Oxford R. L. (2016). “Toward a psychology of well-being for language learners: the “EMPATHICS” vision” in Positive Psychology in SLA. eds. MacIntyre P. Gregersen T. Mercer S. (Bristol, UK: Multilingual Matters), 10–87.

[ref72] Oxford R. L. (2018). “Empathics: a complex dynamic systems (CDS) vision of language learner well-being” in The TESOL Encyclopedia of English Language Teaching. eds. Liontas J. I. Carpini M. D.. doi: 10.1002/9781118784235.eelt0953

[ref73] Oxford R. L. Cuéllar L. (2014). Positive psychology in cross-cultural narratives: Mexican students discover themselves while learning Chinese. Special issue on positive psychology in SLA. Stud. Second Lang. Learn. Teach. 4, 173–203. doi: 10.14746/ssllt.2014.4.2.3

[ref74] Pekrun R. Elliot A. J. Maier M. A. (2006). Achievement goals and discrete achievement emotions: a theoretical model and prospective test. J. Educ. Psychol. 98, 583–597. doi: 10.1037/0022-0663.98.3.58310.1037/0022-0663.98.3.583

[ref76] Peterson C. Seligman M. E. (2004). Character Strengths and Virtues: A Handbook and Classification. Vol. 1. Oxford: Oxford University Press.

[ref77] Piasecka L. (2016). “Activating character strengths through poetic encounters in a foreign language—a case study” in Positive Psychology Perspectives on Foreign Language Learning and Teaching. eds. Gabryś-Barker D. Gałajda D. (Switzerland: Springer International Publishing AG), 75–92.

[ref78] Plonsky L. Ghanbar H. (2018). Multiple regression in L2 research: a methodological synthesis and guide to interpreting R2 values. Mod. Lang. J. 102, 713–731. doi: 10.1111/modl.12509

[ref79] Plonsky L. Oswald F. L. (2014). How big is “big”? Interpreting effect sizes in L2 research. Lang. Learn. 64, 878–912. doi: 10.1111/lang.12079

[ref80] Reyes M. R. Brackett M. A. Rivers S. E. White M. Salovey P. (2012). Classroom emotional climate, student engagement, and academic achievement. J. Educ. Psychol. 104, 700–712. doi: 10.1037/a0027268

[ref81] Seligman M. E. P. (2002). Authentic Happiness: Using the New Positive Psychology to Realize Your Potential for Lasting Fulfillment. New York: Free Press.

[ref82] Seligman M. E. (2012). Flourish: A Visionary New Understanding of Happiness and Well-being. New York, NY: Atria Paperback.

[ref83] Seligman M. (2018). PERMA and the building blocks of well-being. J. Posit. Psychol. 13, 333–335. doi: 10.1080/17439760.2018.1437466

[ref84] Seligman M. E. P. Csikszentmihalyi M. (2000). Positive psychology: an introduction. Am. Psychol. 55, 5–14. doi: 10.1037/0003-066X.55.1.5 11392865

[ref85] Seligman M. E. P. Ernstb R. M. Gillhamc J. Reivicha K. Linkinsd M. (2009). Positive education: positive psychology and classroom interventions. Oxf. Rev. Educ. 35, 293–311. doi: 10.1080/03054980902934563

[ref86] Seligman M. E. P. Steen T. A. Park N. Peterson C. (2005). Positive psychology Progress: empirical validation of interventions. Am. Psychol. 60, 410–421. doi: 10.1037/0003-066X.60.5.410, PMID: 16045394

[ref88] Suh E. Diener E. Fujita F. (1996). Events and subjective well-being: only recent events matter: erratum. J. Pers. Soc. Psychol. 71:842. doi: 10.1037/0022-3514.71.5.842 8656337

[ref89] Tennant R. Hiller L. Fishwick R. Platt S. Joseph S. Weich S.. (2007). The Warwick-Edinburgh mental well-being scale (WEMWBS): development and UK validation. Health Qual. Life Outcomes 5. doi: 10.1186/1477-7525-5-63 PMC2222612 18042300

[ref90] Wang Y. Derakhshan A. Zhang L. J. (2021). Researching and practicing positive psychology in second/foreign language learning and teaching: the past, current status and future directions. Front. Psychol. 12:731721. doi: 10.3389/fpsyg.2021.731721, PMID: 34489835 PMC8417049

[ref91] Yerlikaya E. E. İnanç B. (2007). “Algılanan stres ölçeğinin (ASÖ) Türkçe çevirisinin psikometrik özellikleri” in IX. Ulusal Psikolojik Danışma ve Rehberlik Kongresi (İzmir, Turkey), 274–276.

[ref92] Zika S. Chamberlain K. (1987). Relation of hassles and personality to subjective well-being. J. Pers. Soc. Psychol. 53, 155–162. doi: 10.1037/0022-3514.53.1.155

